# Changes in dynamic arterial elastance induced by volume expansion and vasopressor in the operating room: a prospective bicentre study

**DOI:** 10.1186/s13613-019-0588-6

**Published:** 2019-10-11

**Authors:** Hugues de Courson, Philippe Boyer, Romain Grobost, Romain Lanchon, Musa Sesay, Karine Nouette-Gaulain, Emmanuel Futier, Matthieu Biais

**Affiliations:** 1 Department of Anesthesiology and Critical Care, Pellegrin Bordeaux University Hospital, 33000 Bordeaux, France; 2 Department of Anesthesiology and Critical Care, Clermont-Ferrand University Hospital, 63003 Clermont-Ferrand Cedex 1, France; 3 INSERM, U12-11, Laboratoire de Maladies Rares: Génétique et Métabolisme (MRGM), Bordeaux, France; 4 Équipe R2D2 EA-7281/Faculté de Médecine/Université d’Auvergne, University of Clermont-Ferrand, 63000 Clermont-Ferrand, France; 5 INSERM, U1034, Biology of Cardiovascular Diseases, 33600 Pessac, France

**Keywords:** Arterial pressure, Cardiac output, Doppler

## Abstract

**Background:**

Dynamic arterial elastance (Eadyn), defined as the ratio between pulse pressure variations and stroke volume variations, has been proposed to assess functional arterial load. We evaluated the evolution of Eadyn during volume expansion and the effects of neosynephrine infusion in hypotensive and preload-responsive patients.

**Methods:**

In this prospective bicentre study, we included 56 mechanically ventilated patients in the operating room. Each patient had volume expansion and neosynephrine infusion. Stroke volume and stroke volume variations were obtained using esophageal Doppler, and pulse pressure variations were measured through the arterial line. Pressure response to volume expansion was defined as an increase in mean arterial pressure (MAP) ≥ 10%.

**Results:**

Twenty-one patients were pressure responders to volume expansion. Volume expansion induced a decrease in Eadyn (from 0.69 [0.58–0.85] to 0.59 [0.42–0.77]) related to a decrease in pulse pressure variations more pronounced than the decrease in stroke volume variations. Baseline and changes in Eadyn after volume expansion were related to age, history of arterial hypertension, net arterial compliance and effective arterial elastance. Eadyn value before volume expansion > 0.65 predicted a MAP increase ≥ 10% with a sensitivity of 76% (95% CI 53–92%) and a specificity of 60% (95% CI 42–76%). Neosynephrine infusion induced a decrease in Eadyn (from 0.67 [0.48–0.80] to 0.54 [0.37–0.68]) related to a decrease in pulse pressure variations more pronounced than the decrease in stroke volume variations. Baseline and changes in Eadyn after neosynephrine infusion were only related to heart rate.

**Conclusion:**

Eadyn is a potential sensitive marker of arterial tone changes following vasopressor infusion.

## Introduction

Many recent studies have underlined the point that perioperative hypotension could lead to worse patient outcome mainly because of myocardial injury, cerebrovascular disease and acute kidney injury [1–3]. Therefore, understanding the origins of perioperative hypotension and its prevention is essential to proposing a judicious treatment modality. Basically, the two principal strategies to correct perioperative hypotension are vasopressor infusion and/or volume expansion. During the last decade, many studies tested different indices and their abilities to predict an increase in stroke volume following fluid administration and/or vasopressor infusion [4–6]. Additionally, the prediction of an increase in arterial pressure following volume expansion has been widely studied.

Several years ago, Pinsky et al. proposed a pragmatic, easy-to-use and physiologically robust approach of dynamic arterial elastance [7]. Schematically, arterial elastance (Ea) is defined as the ratio of changes in pressure to changes in volume. Dynamic arterial elastance (Eadyn) could therefore be assessed using the ratio between respiratory-induced changes in arterial pulse pressure and respiratory-induced changes in stroke volume during a single respiratory cycle. Several studies investigated the clinical utility of Eadyn. The most robust data concern the evolution of Eadyn as an indicator of decrease in arterial pressure following a reduction in norepinephrine dosage in septic shock patients [8]. A randomized control trial demonstrated that a therapeutic protocol based on Eadyn may be an efficient guide to decrease norepinephrine infusion in patients following cardiac surgery [9]. Other studies investigated the ability of Eadyn to predict an increase in arterial pressure following volume expansion with discordant results [10–15]. Results of studies including ICU septic patients with acute circulatory failure and receiving vasopressors are very promising [13, 16, 17]. On the another hand, studies including operating room patients without vasopressor support and suffering from non-septic hypovolemia showed conflicting results [11, 12, 15, 18]. We can hypothesize that volume expansion induces different effects on arterial compliance in patients receiving vasopressors or not. Hence, it affects Eadyn in a different way. The very rare studies carried out in the operating theater included very specific patients (liver failure or laparoscopy). Before proposing the use of Eadyn to guide low blood pressure treatment in the operating room, it would be necessary to perform a prospective study on patients, under general anesthesia and are not receiving vasopressors.

The objectives of this bicentre study were to (i) describe Eadyn variations induced by volume expansion and vasopressor infusion, (ii) describe the relationship between baseline Eadyn and the increase in arterial pressure induced by volume expansion and vasopressor infusion and (iii) test the ability of baseline Eadyn to predict an increase in arterial pressure following volume expansion in the operating room.

## Materials and methods

### Patients

This prospective, bicentre study was approved by the Institutional Review Board (Comité de Protection des Personnes Sud-Ouest et Outre Mer III, Bordeaux, France N°DC2016/125) and was registered at French National Commission for Data Protection and Liberties (CNIL N°1980317).

Fifty-six non-consecutive patients were included after oral informed consent. (Written informed consent was waived by the Institutional Review Board.) Inclusion criteria were patients scheduled for neurosurgery or elective abdominal surgery, older than 18 years, equipped with radial arterial catheter and esophageal Doppler (CardioQ ODM+, Deltex Medical, Chichester, UK) for cardiac output monitoring. Non-inclusion criteria were preoperative lung disease, intracranial hypertension, left ventricular ejection fraction below 50%, arrhythmia, suspected right ventricular dysfunction, extreme body weight (BMI > 40 or < 15 kg/m^2^).

### Perioperative management

Standard monitoring included noninvasive blood pressure, heart rate, peripheral oxygen saturation and continuous electrocardiography. After preoxygenation, anesthesia was induced using propofol and remifentanil or sufentanil. Propofol or sevoflurane and remifentanil or sufentanil were used for maintenance of anesthesia. Following tracheal intubation, patient’s lungs were ventilated with a mixture of air/oxygen using volume control mode. Tidal volume was set between 6 and 8 ml/kg of ideal body weight, and positive end-expiratory pressure was set between 6 and 10 cmH_2_O (Primus, Dräger, Lübeck, Germany, or Avance, General Electric Healthcare, Helsinki, Finland). Peripheral oxygen saturation was maintained above 96%, and the respiratory rate was adjusted to maintain end-tidal carbon dioxide concentration between 30 and 35 mmHg. The inspiratory-to-expiratory ratio was set to 1/2.

### Hemodynamic monitoring

All patients were equipped with a radial arterial catheter inserted just after the induction of anesthesia (Vygon, Ecouen, France). The catheter was connected to a bedside monitor (Spacelabs Healthcare Company Headquarters, Issaquah, WA, USA, or IntelliVue MP70, Philips Healthcare, Andover, MA, USA) for mean arterial pressure (MAP) and pulse pressure variation (PPV) monitoring. After tracheal intubation, the probe was inserted into the esophagus via the nasal route and the good quality of the signal was confirmed as previously described.

PPV was derived from the bedside monitor by manual calculation of the difference between systolic and diastolic blood pressure. The maximal (Pulse Pressure max) and minimal (Pulse Pressure min) differences were determined during three consecutive respiratory cycles. The mean values of the three measurements were used to calculate arterial pulse pressure variability: PPV = (Pulse Pressure max − Pulse Pressure min)/[(Pulse Pressure max + Pulse Pressure min)/2] × 100, as previously described [19]. PPV was measured directly on the monitor using a screenshot.

Stroke volume, cardiac output, stroke volume variation (SVV), corrected flow time and peak velocity of aortic blood flow were assessed using esophageal Doppler (CardioQ ODM+, Deltex Medical, Gamida, Eaubonne France). SVV was calculated automatically as follows: (Stroke Volume max − Stroke Volume min)/[(Stroke Volume max + Stroke Volume min)/2] over one respiratory cycle. The SVV value was averaged over five respiratory cycles [20]. SVV value was recorded immediately after the measurement of PPV in order to record two values that covered the same time period.

Eadyn was calculated as the ratio between PPV and SVV. Net arterial compliance (*C*) was calculated as the ratio between stroke volume and pulse pressure [21]. Arterial resistance (*R*) was calculated as the ratio between mean arterial pressure and cardiac output. Arterial elastance was calculated using these two formulas: Ea_SAP_ = (systolic arterial pressure × 0.9)/stroke volume and Ea_MAP_ = mean arterial pressure/stroke volume [22, 23].

### Study design

Measurements were performed in the operating room, between the end of induction of anesthesia and the end of surgery. Volume expansions and neosynephrine infusions were performed according to the routine care of the patients. Each patient received volume expansion before neosynephrine infusion. If several volume expansions or neosynephrine infusions were done in one patient, only the first volume expansion or the first neosynephrine infusion was recorded.

Volume expansion was performed if MAP ≤ 65 mmHg and SVV > 10%, and neosynephrine was infused if MAP ≤ 65 mmHg regardless of the SVV value, on the discretion of the physician in charge.

Volume expansion was done with 250 ml 0.9% saline over 10 min. One set of measurements was performed immediately before volume expansion, and the second set was done 2–3 min after the end of fluid administration.

Vasopressor infusion consisted of a fixed dose of 50 mcg of neosynephrine. One set of measurements was performed immediately before vasopressor infusion, and the second set was performed 2 to 3 min after the infusion when MAP was stabilized (MAP variation below 5% during 1 min).

Patients with hemodynamic instability requiring a decrease (or an increase) in anesthesia drug dosage, fluid infusion or administration of vasopressors other than in the protocol were excluded. Another exclusion criterion was any change in ventilatory setting by the physician in charge of the patient.

### Statistical analysis

Data are expressed as median [percentile, 25–75] or mean ± SD where appropriate. Normality of the distribution was tested using D’Agostino–Pearson test. Pressure response to volume expansion was defined as an increase in MAP ≥ 10% [13, 14]. The effects of neosynephrine and volume expansion on hemodynamic parameters were analyzed using Wilcoxon rank sum test. Mann–Whitney test was used to compare hemodynamic variables before fluid challenge or neosynephrine infusion in pressure non-responder and responder patients. The relationship between Eadyn and changes in MAP induced by volume expansion was tested using Spearman rank test.

The receiver-operating characteristic (ROC) curves were generated for Eadyn and MAP to test their abilities to predict pressure response to volume expansion. Area under the receiver-operating characteristic curves were compared using De Long test [24]. The best threshold values were identified using the Youden Index (specificity + sensitivity − 1). The gray zone was determined as follows: The low cutoff value was defined to exclude positive fluid challenge in 90% of patients, whereas the high cutoff value was defined to predict positive fluid challenge in 90% of cases [25]. A diagnostic test is considered to have good accuracy when its area under the ROC curve is ≥ 0.75 [26]. Fifty-six patients were needed to demonstrate the ability of Eadyn to predict pressure responsiveness with good accuracy, i.e., area under the ROC curve > 0.75 (ratio of pressure responders = 1/3, null hypothesis = 0.50, type I error of 5% and type II error of 10%).

Random effects models were estimated in order to study the effect of several covariates on the temporal evolution of Eadyn, SVV and PPV during fluid challenge and neosynephrine infusion.

For each hemodynamic maneuver (i.e., fluid challenge and neosynephrine infusion), two different models were estimated for Eadyn, PPV and SVV. The first model included covariates related to arterial load and the second cardiac covariates. Each model was a fully adjusted model including all covariates of the study. Only baseline values (before hemodynamic maneuver) were included. To study the effect of covariates on temporal evolution of Eadyn, PPV and SVV, random effects models were estimated with subjects being considered as the random factors. For each random effects model, because only two repeated measurements were performed, we considered time function as linear. Hence, we estimated the fixed intercept, fixed effects, random intercept and time interaction between time and each covariate. The contribution of each covariate on temporal evolution was tested by a Wald test on the time–covariate interaction term [27].

Statistical analysis was performed using Medcalc (software 11.6; Mariakerke, Belgium) and R Development Core Team ([2008]. R: A language and environment for statistical computing; R Foundation for Statistical Computing, Vienna, Austria. ISBN 3-900051-07-0, URL).

## Results

### Patient characteristics

**Fig. 1:**
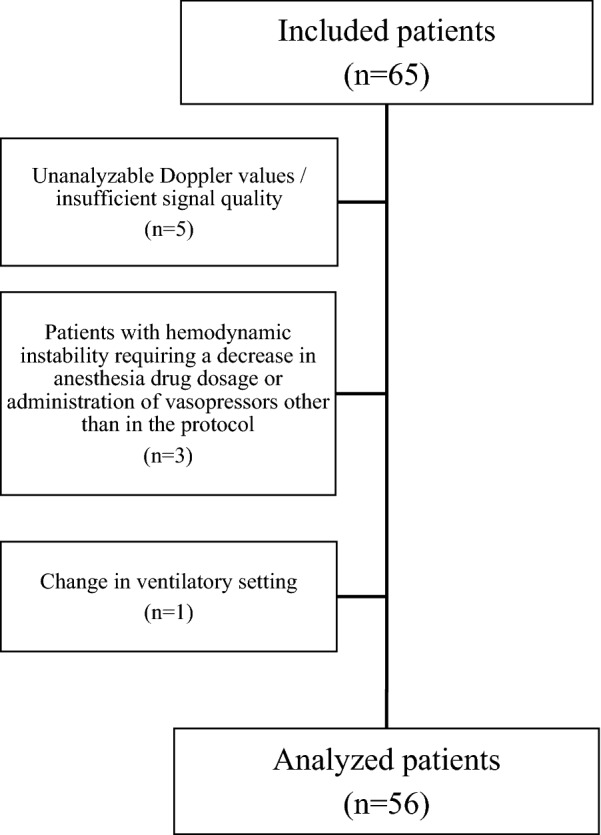
Flowchart of patients screened and included in the study

Fifty-six patients were included (Fig. 1). Patient characteristics are shown in Table 1. Hemodynamic variables are shown in Table 2. The distribution of Eadyn was not normal. Each patient received volume expansion before neosynephrine infusion.

**Table 1:** Main characteristics of patients (*n* = 56)

Characteristics	Overall *N* = 56	Bordeaux *N* = 43	Clermont *N* = 13
Age (years)	57 ± 13	54 ± 14	60 ± 11
Sex, M/F (*n*)	21/35	16/27	5/8
Height (cm)	168 ± 9	169 ± 9	169 ± 11
Weight (kg)	72 ± 15	73 ± 15	67 ± 16
Ideal body weight (kg)	62 ± 10	62 ± 9	63 ± 11
Tidal volume (ml)	455 ± 66	441 ± 55	500 ± 79
Tidal volume (ml/kg of ideal body weight)	7.4 ± 0.8	7.2 ± 0.7	8.0 ± 0.7
Respiratory rate (cycles/min)	13 ± 1	13 ± 1	12 ± 1
Positive end-expiratory pressure (cmH_2_O)	5 [5–6]	5 [5–6]	6 [5–8]
F_I_O_2_ (%)	45 [40–50]	45 [40–50]	45 [40–45]
Driving pressure (cmH_2_O)	11 ± 3	11 ± 3	8 ± 2
ASA status I/II/III	7/16/33	5/9/29	2/7/4
Surgery (*n*)			
Neurosurgery	43	43	0
Abdominal surgery	13	0	13
Comorbidities			
Arterial hypertension	17	16	1
Smoking	5	4	1
Diabetes	4	4	0
Stroke	2	2	0
Medications			
ACE inhibitors	9	7	2
Statins	5	5	0
Calcium blockers	5	4	1
ARB	5	4	1
Antidiabetic drug	3	3	0
Beta-blockers	2	2	0

Values are mean ± SD, or median [percentile 25–75] or number (*n*)

*F*_*I*_*O*_*2*_ inspired oxygen fraction, *ACE* angiotensin-converting enzyme, *ARB* angiotensin II receptor blocker

**Table 2:** Hemodynamic variables before and after volume expansion/neosynephrine infusion

	Before VE	After VE	*P*1	Before NEO	After NEO	*P*2
Heart rate (bpm)	64 [58–74]	60 [55–71]	< 0.0001	60 [56–71]	57 [52–65]	< 0.0001
SAP (mmHg)	80 [75–86]	83 [78–89]	0.006	80 [77–87]	102 [93–115]	< 0.0001
MAP (mmHg)	59 [53–64]	63 [59–66]	< 0.0001	60 [55–64]	75 [69–85]	0.0001
DAP (mmHg)	47 [43–50]	48 [43–52]	0.04	48 [43–51]	59 [54–65]	< 0.0001
PP (mmHg)	32 [28–38]	35 [30–40]	0.004	34 [29–39]	44 [36–52]	< 0.0001
Stroke volume (ml)	64 [54–81]	76 [63–97]	< 0.0001	81 [66–96]	64 [55–82]	< 0.0001
Cardiac output (l/min)	4.4 [3.5–5.3]	4.9 [3.7–6.2]	< 0.0001	4.9 [4.0–6.1]	3.8 [2.9–5.0]	< 0.0001
PPV (%)	12 [8–14]	8 [6–10]	< 0.0001	9 [6–11]	6 [4–9]	< 0.0001
SVV (%)	16 [12–22]	14 [11–17]	< 0.0001	14 [10–16]	11 [8–15]	0.0005
Eadyn	0.69 [0.58–0.85]	0.59 [0.42–0.77]	0.0002	0.67 [0.48–0.80]	0.54 [0.37–0.68]	< 0.0001
Ea (mmHg/ml)/SAP	1.06 [0.89–1.34]	0.91 [0.77–1.16]	< 0.0001	0.90 [0.77–1.12]	1.42 [1.08–1.88]	< 0.0001
Ea (mmHg/ml)/MAP	0.92 [0.69–1.11]	0.80 [0.63–0.99]	< 0.0001	0.72 [0.60–0.93]	1.09 [0.89–1.53]	< 0.0001
C (ml/mmHg)	2.11 [1.67–2.46]	2.33 [1.70–2.81]	0.001	2.37 [1.96–2.81]	1.57 [1.14–1.94]	< 0.0001
R (mmHg/s/ml)	13.3 [10.8–16.9]	12.8 [9.6–17.3]	0.01	12.1 [9.4–14.9]	20.5 [13.7–28.1]	< 0.0001
FTc (ms)	305 [269–333]	323 [291–354]	< 0.0001	326 [267–351]	303 [280–333]	< 0.0001
PV (cm/s)	69 [58–89]	72 [62–91]	0.0004	75 [62–94]	64 [54–77]	< 0.0001

Values are median [percentile, 25–75]

*C* net arterial compliance, *DAP* diastolic arterial pressure, *Ea* arterial elastance, *Eadyn* dynamic arterial elastance, *FTc* corrected aortic flow time, *MAP* mean arterial pressure, *VE* volume expansion, *NEO* neosynephrine, *PP* arterial pulse pressure, *PPV* pulse pressure variations, *PV* peak velocity of aortic blood flow, *R* arterial resistance, *SAP* systolic arterial pressure, *SVV* stroke volume variations, P1 *p* value of the difference obtained before and after volume expansion, P2 *p* value of the difference obtained before and after neosynephrine infusion

### Effects of volume expansion

**Fig. 2:**
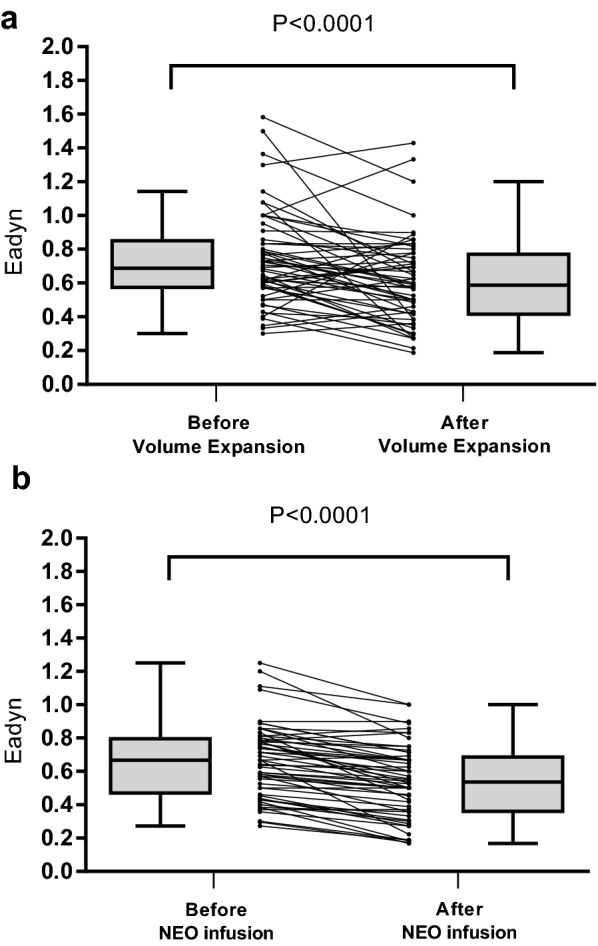
**a** Box plot (median values, inter-quartile range) and individual values of dynamic arterial elastance (Eadyn) before and after volume expansion. **b** Box plot (median values, inter-quartile range) and individual values of dynamic arterial elastance (Eadyn) before and after neosynephrine (NEO) infusion

Volume expansion induced a significant increase in arterial pressure (systolic, diastolic, mean and pulse pressure), stroke volume (16% [10–25%]), cardiac output (14% [10–18%]) and net arterial compliance associated with a significant decrease in heart rate, PPV, SVV, Eadyn, Ea and *R* (Table 2 and Fig. 2a). Forty-five patients presented an increase in stroke volume ≥ 10%. Volume expansion induced a significantly larger decrease in PPV than in SVV: (− 27% (− 48 to − 13%) versus − 15% (− 29 to 0%), respectively), *p* = 0.0002. Baseline and changes in Eadyn after volume expansion were related to age, arterial hypertension, compliance and effective arterial elastance (Tables 3, 4). After volume expansion, Eadyn decreased in 38 patients (68%), remained unchanged in 2 patients (4%) and increased in 16 patients (29%). Twenty-one patients were pressure responders to volume expansion. Prior to volume expansion, Eadyn was higher and MAP lower in responder than in non-responder patients (Table 5). Volume expansion induced different pressure effects in responders and non-responder patient. Ea, *C* and *R* were similar after volume expansion in pressure responders, whereas Ea and *R* decreased and *C* increased in non-responder patients (Table 5).

**Table 3:** Random effects models for studying factors associated with temporal evolution of Eadyn, PPV and SVV during fluid challenge

	Eadyn				PPV				SVV			
	Baseline effect	*p* value	Time course effect	*p* value	Baseline effect	*p* value	Time course effect	*p* value	Baseline effect	*P* value	Time course effect	*P* value
Arterial load												
Intercept	− 1.383	–	–	–	− 17.310	–	–	–	6.989	–	–	–
Age (for 1 year increase)	0.011	0.018*	− 0.007	0.010*	0.168	0.028*	− 0.101	0.023*	0.068	0.468	− 0.044	0.336
Arterial hypertension (yes vs. no)	− 0.301	0.028*	0.156	0.041*	0.899	0.678	− 0.165	0.895	5.781	0.035*	− 2.675	0.046*
Ea (for 1 mmHg/ml increase)	0.799	0.038*	− 0.302	0.156	18.286	0.004*	− 6.048	0.089	11.630	0.130	− 3.035	0.414
Compliance (for 1 ml/mmHg increase)	0.457	0.002*	− 0.205	0.013*	4.976	0.034*	− 2.053	0.128	0.356	0.902	− 0.382	0.786
Resistance (for 1 mmHg/s/ml increase)	− 0.013	0.459	0.005	0.646	− 0.499	0.084	0.189	0.255	− 0.406	0.259	0.105	0.545
Cardiac covariates												
Intercept	1.523	–	–	–	25.200	–	–	–	17.112	–	–	–
HR (for 1 beat/min increase)	0.007	0.137	− 0.002	0.356	0.116	0.095	− 0.051	0.203	0.053	0.544	− 0.026	0.544
FTc (for 1 ms increase)	− 0.004	0.032*	0.002	0.107	− 0.016	0.540	0.009	0.525	0.052	0.109	− 0.012	0.436
Stroke volume (for 1 mmHg increase)	0.003	0.334	− 0.002	0.435	− 0.055	0.308	− 0.007	0.826	− 0.100	0.141	− 0.006	0.847
PV	− 0.003	0.386	0.001	0.498	− 0.109	0.052	0.059	0.070	− 0.123	0.078	0.007	0.037*

Results are presented for fully adjusted models with all studied covariates included. *p* value represents the *p* value of Wald test for covariate–time interaction estimator

*p value < 0.05 means statistical significance

*Ea* arterial elastance, *FTc* corrected aortic flow time, *HR* heart rate, *PV* peak velocity of aortic blood flow

**Table 4:** Random effects models for studying factors associated with temporal evolution of Eadyn, PPV and SVV during neosynephrine infusion

	Eadyn				PPV				SVV			
	Baseline effect	*p* value	Time course effect	*p* value	Baseline effect	*p* value	Time course effect	*p* value	Baseline effect	*p* value	Time course effect	*p* value
Arterial load												
Intercept	0.320	–	–	–	5.402	–	–	–	0.939	–	–	–
Age (for 1 year increase)	− 0.002	0.761	0.003	0.275	− 0.004	0.933	0.014	0.562	− 0.032	0.722	0.021	0.627
Arterial hypertension (yes vs. no)	0.031	0.856	− 0.052	0.484	0.968	0.453	− 0.611	0.346	2.406	0.331	− 1.172	0.328
Ea (for 1 mmHg/ml increase)	− 0.237	0.704	0.190	0.480	9.265	0.049*	− 0.377	0.871	16.440	0.068	− 1.409	0.744
Compliance (for 1 ml/mmHg increase)	− 0.084	0.647	0.050	0.530	0.421	0.760	0.424	0.539	2.269	0.390	− 0.448	0.725
Resistance (for 1 mmHg/s/ml increase)	− 0.002	0.950	0.002	0.897	− 0.330	0.114	0.070	0.500	− 0.450	0.260	0.019	0.920
Cardiac covariates												
Intercept	0.958	–	–	–	14.185	–	–	–	13.030	–	–	–
HR (for 1 beat/min increase)	0.006	0.047*	− 0.001	0.534	0.024	0.607	0.015	0.510	− 0.064	0.490	0.029	0.513
FTc (for 1 ms increase)	− 0.002	0.092	0.001	0.874	0.002	0.913	− 0.005	0.542	0.042	0.200	− 0.001	0.950
Stroke volume (for 1 mmHg increase)	− 0.001	0.927	− 0.002	0.754	− 0.096	0.001*	0.032	0.031*	− 0.135	0.019*	0.024	0.382
PV	0.001	0.780	− 0.001	0.912	0.036	0.147	− 0.021	0.088	0.056	0.249	− 0.043	0.070

Results are presented for fully adjusted models with all studied covariates included. *p* value represents the *p* value of Wald test for covariate–time interaction estimator

*p value < 0.05 means statistical significance

*Ea* arterial elastance, *FTc* corrected aortic flow time, *HR* heart rate, *PV* peak velocity of aortic blood flow

**Table 5:** Hemodynamic variables before and after volume expansion in responder and non-responder patients

	Responders (*n* = 21)			Non-responders (*n* = 35)			
	Before VE	After VE	*P*1	Before VE	After VE	*P*2	*P*3
Heart rate (bpm)	62 [58–72]	60 [54–71]	0.0028	65 [57–74]	60 [56–71]	< 0.0001	0.71
SAP (mmHg)	77 [66–80]	86 [79–93]	0.0001	82 [79–87]	80 [77–86]	0.49	0.0011
MAP (mmHg)	54 [51–59]	62 [60–66]	0.0001	65 [57–64]	69 [58–65]	< 0.0001	0.006
DAP (mmHg)	43 [41–49]	50 [45–53]	0.0001	47 [43–51]	48 [41–52]	0.16	0.03
Stroke volume (ml)	60 [53–73]	72 [62–85]	0.0006	64 [55–92]	81 [67–97]	< 0.0001	0.17
Cardiac output (l/min)	4.0 [3.3–4.6]	4.3 [3.5–5.7]	0.0005	4.6 [3.6–5.6]	5.4 [3.7–6.5]	< 0.0001	0.10
PPV (%)	14 [11–19]	8 [7–11]	0.0001	10 [8–13]	8 [6–10]	0.0001	0.001
SVV (%)	16 [13–23]	14 [13–19]	0.0017	15 [12–21]	13 [10–16]	0.0006	0.46
Eadyn	0.75 [0.66–1.02]	0.57 [0.48–0.70]	0.0002	0.62 [0.48–0.82]	0.63 [0.39–0.82]	0.14	0.01
Ea (mmHg/ml)/SAP	1.06 [0.90–1.30]	1.06 [0.86–1.27]	0.34	1.06 [0.84–1.36]	0.88 [0.73–1.11]	< 0.0001	0.96
Ea (mmHg/ml)/MAP	0.93 [0.69–1.1]	0.92 [0.70–0.97]	0.20	0.85 [0.67–1.12]	0.74 [0.60–1.00]	< 0.0001	0.89
C (ml/mmHg)	2.15 [1.66–2.37]	2.02 [1.60–2.52]	0.30	2.09 [1.70–2.46]	2.38 [1.95–2.94]	< 0.0001	0.91
R (mmHg/s/ml)	13.4 [10.5–18.0]	14.7 [9.6–20.1]	0.7	13.0 [10.9–16.5]	12.5 [9.4–17.0]	0.001	0.81
FTc (ms)	292 [266–306]	323 [298–334]	0.0002	317 [272–346]	331 [285–380]	0.0001	0.02
PV (cm/s)	66 [56–84]	66 [56–85]	0.03	75 [59–91]	76 [66–94]	0.005	0.30

Values are expressed as median [percentile, 25–75]

*P1* difference between before and after VE in responders, *P2* difference between before and after VE in non-responders, *P3* difference between responders and non-responders before volume expansion, *responders* MAP increase ≥ 10% after volume expansion, *C* net arterial compliance, *DAP* diastolic arterial pressure, *Ea* effective arterial elastance, *Eadyn* dynamic arterial elastance, *FTc* corrected aortic flow time, *MAP* mean arterial pressure, *VE* volume expansion, *NEO* neosynephrine, *PP* arterial pulse pressure, *PPV* pulse pressure variations, *PV* peak velocity of aortic blood flow, *R* arterial resistance, *SAP* systolic arterial pressure, *SVV* stroke volume variations

### Effects of neosynephrine infusion

Neosynephrine infusion induced a decrease in heart rate, stroke volume, PPV, SVV, Eadyn and *C* and an increase in arterial pressure (systolic, diastolic, mean and pulse pressure), Ea and *R* (Fig. 2b) and an increase in MAP (Table 2). Neosynephrine infusion induced a significantly larger decrease in PPV than in SVV: (− 31% (− 40 to − 18%)) versus − 14% (− 28 to 0%), respectively), *p* < 0.0001. Baseline and changes in Eadyn after neosynephrine infusion were only related to baseline heart rate (Tables 3, 4).

### Relationship between Eadyn and MAP

We observed a weak correlation between baseline Eadyn and the increase in MAP induced by volume expansion (*r*^2^ = 0.11; *p* = 0.01). However, there was neither any correlation between baseline Eadyn and baseline MAP values (*p* = 0.1) nor between changes in Eadyn and the increase in MAP associated with volume expansion.

Concerning neosynephrine infusion, there was no correlation between baseline Eadyn and baseline MAP values (*p* = 0.3), between baseline Eadyn and neosynephrine-induced increase in MAP (*p* = 0.76) and between neosynephrine-induced changes in Eadyn and neosynephrine-induced changes in MAP (*p* = 0.88).

### Prediction of pressure response to volume expansion

**Fig. 3:**
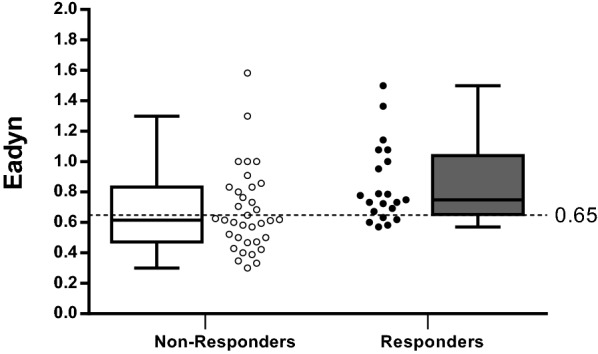
Box plot (median values, inter-quartile range) and individual values of dynamic arterial elastance (Eadyn) before and after volume expansion in pressure responders (volume expansion induced increase in mean arterial pressure ≥ 10%) and pressure non-responders

**Fig. 4:**
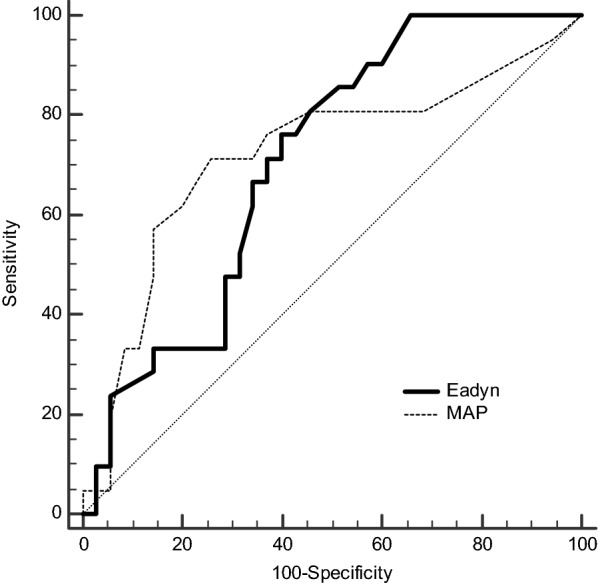
Receiver-operating characteristic curves showing the ability of baseline dynamic arterial elastance (Eadyn) and baseline mean arterial pressure (MAP) to predict an increase in MAP ≥ 10% following volume expansion

Eadyn values before volume expansion in pressure responders and non-responders are shown in Fig. 3. Baseline Eadyn value > 0.65 before volume expansion predicted an increase ≥ 10% in MAP with a sensitivity of 76% (95% CI 53–92%) and a specificity of 60% (95% CI 42–76%) (Fig. 4). The baseline MAP value ≤ 57 mmHg predicted an increase ≥ 10% in MAP with a sensitivity of 71% (95% CI 48–89%) and a specificity of 74% (95% CI 57–88%). Gray zones ranged from 0.59 to 0.98 for Eadyn (included 52% of patients) and from 52 to 64 mmHg for MAP (including 77% of patients). The area under the ROC curve generated for baseline Eadyn (0.71, 95% CI 0.57 to 0.84) was similar to the one generated for baseline MAP (0.72, 95% CI 0.57–0.87), *p* = 0.89 (Fig. 4 and Table 6). Baseline Eadyn value < 0.52 was associated with a negative predictive value of 100% (positive predictive value of 48%).

**Table 6:** Ability to predict the increase in mean arterial pressure ≥ 10% after infusion of 250 ml saline over 10 min

Variable	Best threshold	AUROC (95% CI)	Sensitivity (95% CI)	Specificity (95% CI)	Youden Index *J*	+LR	−LR
Eadyn	> 0.65	0.71 (0.57–0.82)	76 (53–92)	60 (42–76)	0.36	1.9 (1.2–3.1)	0.4 (0.2–0.9)
MAP (mmHg)	≤ 57	0.72 (0.58–0.83)	71 (48–89)	74 (57–88)	0.46	2.8 (1.5–5.2)	0.4 (0.2–0.8)

The best threshold value was determined using Youden Index

Youden Index *J* = Sensitivity + Specificity − 1

*AUROC* area under receiver-operating characteristic curves, *95% CI* 95% confidence interval, *Eadyn* dynamic arterial elastance, *MAP* mean arterial pressure, *+LR* positive likelihood ratio, *−LR* negative likelihood ratio

Eleven patients presented an increase in stroke volume less than 10% (8% [5–9%]). When these eleven volume non-responder patients were excluded, the AUC generated for Eadyn and MAP was not different from the AUC generated with all patients included (0.75, 95% CI 0.60 to 0.87 versus 0.71, 95% CI 0.57 to 0.84 (*p *> 0.05) and 0.75, 95% CI 0.60 to 0.86 versus 0.72, 95% CI 0.57 to 0.87, respectively (*p *> 0.05)). The best threshold values for Eadyn and MAP were also similar.

## Discussion

This bicentre study suggests that in hypotensive and preload-responsive patients (i) both volume expansion and vasopressor infusion induced a decrease in Eadyn, (ii) very poor or no relationship was found between baseline Eadyn and changes in MAP induced by volume expansion or vasopressor infusion and (iii) baseline Eadyn was moderately able to predict the increase in MAP (but not significantly different from MAP baseline values), following volume expansion.

### Ea and Eadyn

The arterial load describes all the extracardiac factors opposing left ventricular ejection [28]. In clinical practice, arterial load is often related to systemic vascular resistance. However, this approach does not take into account the cyclic nature of the blood pressure and flow imposed by cardiac contractions. A more accurate and precise insight of arterial load is the evaluation of aortic input impedance, but it is not feasible at the bedside. Sunagawa proposed that a single variable called effective arterial elastance or Ea could integrate all the components of aortic impedance [29]. A dynamic and functional assessment of arterial load called dynamic arterial elastance has been proposed a few years ago. It represents the dynamic interaction between changes in arterial pressure and stroke volume during a respiratory cycle. Several studies have evaluated this new approach, in particular the ability of Eadyn to predict an increase in arterial pressure following volume expansion. Most of these studies on patients treated with vasopressors revealed discordant results [8–13]. The originality of our study is to include mechanically ventilated patients in the operating room who are not receiving vasopressors which may alter arterial load.

### Effects of volume expansion

The present study shows that in pressure responder patients, volume expansion increases stroke volume and has no effect on arterial load, leading to an increase in arterial pressure. On the other hand, in pressure non-responder patients, volume expansion increases stroke volume associated with vasodilation (shear stress) attested by an increase in arterial compliance and a decrease in resistance. Volume expansion induced a decrease in Eadyn mainly due to a larger decrease in PPV than in SVV. Changes in PPV were related to arterial load covariates, whereas changes in SVV were not. This may be explained by the fact that PPV was measured peripherally in a radial artery and was sensitive to arterial properties, whereas SVV was measured at the level of the descending aorta. We found that both Eadyn and PPV were related to arterial hypertension and age. This finding is consistent with a previous study [18] and is probably related to arterial stiffness.

### Eadyn and vasopressors

Using Eadyn as a physiological marker of arterial tone during vasopressor utilization seems plausible. Vasopressor infusion induces a systematic effect on arterial load which could be assessed by Eadyn. Some studies evaluated the evolution of Eadyn as an indicator of decrease in arterial pressure following a reduction in norepinephrine dosage in septic shock patients [8]. A randomized control trial demonstrated that a therapeutic protocol based on Eadyn may be an efficient guide to decrease norepinephrine infusion in patients following cardiac surgery [9]. In the present study, we observed a significant decrease in Eadyn following neosynephrine infusion in 89% of patients. Neosynephrine infusion induces vasoconstriction (increase in resistance and decrease in compliance) and a decrease in both stroke volume and cardiac output. We observed that both contractility and preload parameters were impacted. The present study was not designed to elucidate this issue. We found that arterial load and cardiac covariates have very few or no effect on the time course of Eadyn, PPV and SVV. This could be explained by the predominant effect of neosynephrine on the rest of the temporal effects.

### Clinical use of Eadyn

Based on our experience, an isolated measurement of Eadyn could not inform the clinician of a possible increase in blood pressure after volume expansion. This remains true, even if we focus only on patients exhibiting an increase in stroke volume ≥ 10% after volume expansion (*n* = 45). Our results are in contradiction with some previous studies [11, 12, 25]. Some of the main factors explaining these differences were the fact that our patients were neither septic nor recipients of vasopressor treatment. This is of major importance because vasopressor infusion can alter arterial load. For example, arterial compliance does not change following volume expansion in patients treated with norepinephrine, whereas it improves after volume expansion in patients free of norepinephrine. Such alterations may explain in part our results. Our findings suggest that Eadyn was not helpful to predict an increase in arterial pressure after volume expansion in mechanically ventilated, preload-responsive and vasopressor-free patients in the operating room.

### Limitations

Our study presents several limitations: First, we did not randomize the order of volume expansion and neosynephrine infusion (all patients received volume expansion before vasopressor infusion) and we only recorded the first volume expansion and the first vasopressor infusion done in each patient. Second, according to local practices, we chose neosynephrine and not norepinephrine despite recent data suggesting the possible superiority of norepinephrine over neosynephrine in patients under general anesthesia in the operating room [30–32]. Third, the two linear mixed models show very different effects of hemodynamic covariates on the time course of Eadyn, PPV and SVV. In the two models, effects remained very low due to a foreseeable lack of power related to a low sample size.

## Conclusion

To conclude, in this bicentre and prospective study, we observed that (i) neosynephrine infusion induced a decrease in Eadyn in a very large proportion of patients, whereas volume expansion induced a decrease in Eadyn in only two-thirds of patients, and (ii) Eadyn was poorly able to predict any increase in mean arterial pressure following volume expansion or neosynephrine infusion. Eadyn seems to be a sensitive marker of arterial tone changes following vasopressor infusion.

## Data Availability

Data are available from the authors on reasonable request.

## Abbreviations

Ea: arterial elastance
Eadyn: dynamic arterial elastance
MAP: mean arterial pressure
PPV: pulse pressure variations
ROC: receiver-operating characteristics
SVV: stroke volume variations
